# Supplementary material for: Mutations in PCYT2 disrupt etherlipid biosynthesis and cause a complex hereditary spastic paraplegia
Source: Brain. 2019 Oct 22;142(11):3382–97. doi: 10.1093/brain/awz291 (PMC6821184; doi:10.1093/brain/awz291)
Supplement: awz291_Supplementary_Data [file awz291_supplementary_data.zip › awz291-Suppl_data/Supplementary_Data4.zip!/Supplementary information 3 - fibroblast lipidomics.zip!/Fibroblast lipidomics/Controle vs Patient2/Statistical plots and data/Heatmap sorted on p-value and FC.pdf]

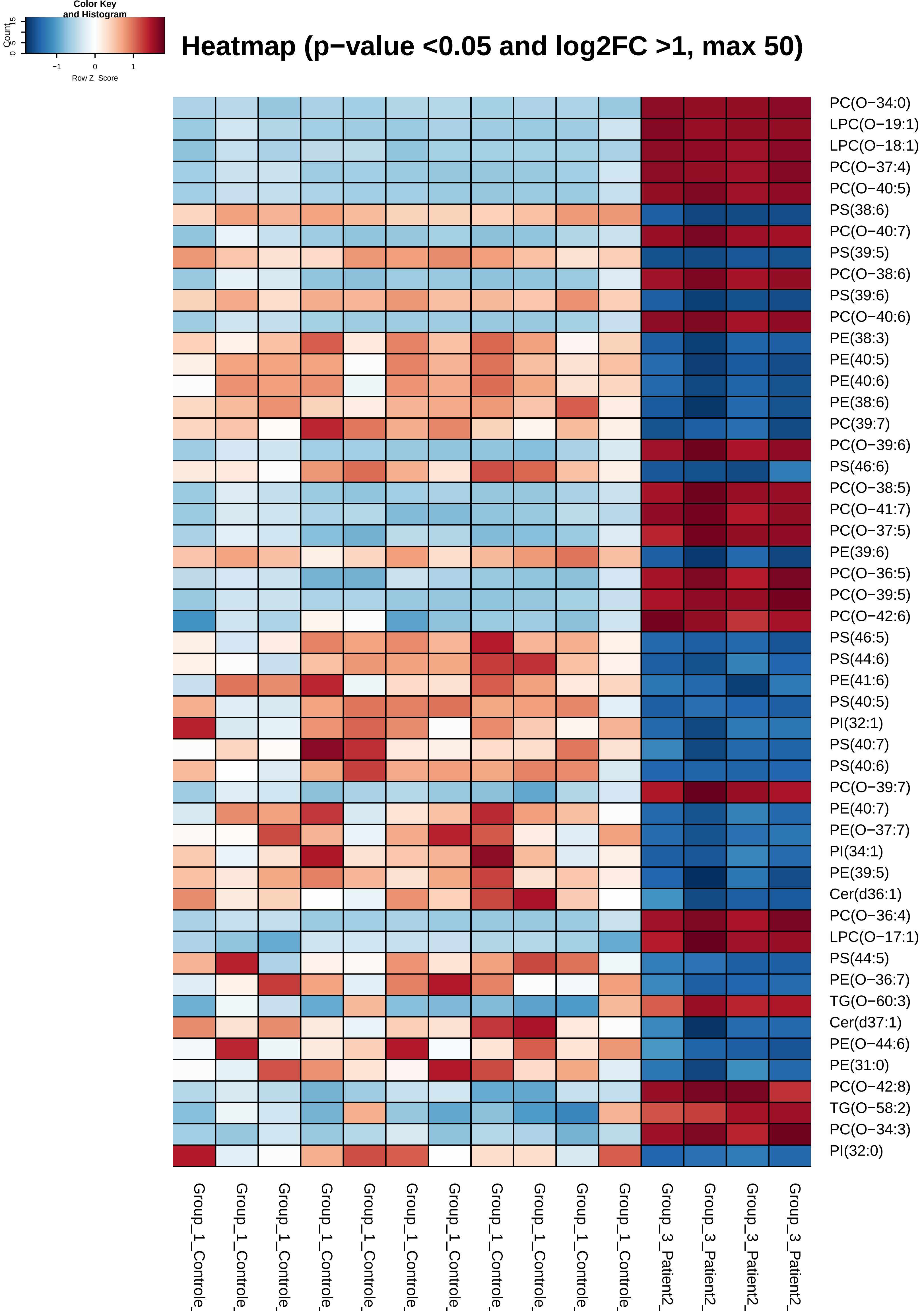

Color Key
and Histogram
Count
0 5 15
−1 0 1
Row Z−Score
Heatmap (p−value <0.05 and log2FC >1, max 50)
PC(O−34:0)
LPC(O−19:1)
LPC(O−18:1)
PC(O−37:4)
PC(O−40:5)
PS(38:6)
PC(O−40:7)
PS(39:5)
PC(O−38:6)
PS(39:6)
PC(O−40:6)
PE(38:3)
PE(40:5)
PE(40:6)
PE(38:6)
PC(39:7)
PC(O−39:6)
PS(46:6)
PC(O−38:5)
PC(O−41:7)
PC(O−37:5)
PE(39:6)
PC(O−36:5)
PC(O−39:5)
PC(O−42:6)
PS(46:5)
PS(44:6)
PE(41:6)
PS(40:5)
PI(32:1)
PS(40:7)
PS(40:6)
PC(O−39:7)
PE(40:7)
PE(O−37:7)
PI(34:1)
PE(39:5)
Cer(d36:1)
PC(O−36:4)
LPC(O−17:1)
PS(44:5)
PE(O−36:7)
TG(O−60:3)
Cer(d37:1)
PE(O−44:6)
PE(31:0)
PC(O−42:8)
TG(O−58:2)
PC(O−34:3)
PI(32:0)
Group_1_Controle_
Group_1_Controle_
Group_1_Controle_
Group_1_Controle_
Group_1_Controle_
Group_1_Controle_
Group_1_Controle_
Group_1_Controle_
Group_1_Controle_
Group_1_Controle_
Group_1_Controle_
Group_3_Patient2_
Group_3_Patient2_
Group_3_Patient2_
Group_3_Patient2_
